# Deep learning for inner speech recognition: a pilot comparative study of EEGNet and a spectro-temporal Transformer on bimodal EEG-fMRI data

**DOI:** 10.3389/fnhum.2025.1668935

**Published:** 2025-10-21

**Authors:** Ahmad H. Milyani, Eyad Talal Attar

**Affiliations:** ^1^Department of Electrical and Computer Engineering, Faculty of Engineering, King Abdulaziz University, Jeddah, Saudi Arabia; ^2^Center of Excellence in Intelligent Engineering Systems (CEIES), King Abdulaziz University, Jeddah, Saudi Arabia

**Keywords:** inner speech, EEG, deep learning, Transformer, EEGNET, brain–computer interface (BCI), neuroprosthetics, imagined speech

## Abstract

**Background:**

Inner speech—the covert articulation of words in one’s mind—is a fundamental phenomenon in human cognition with growing interest across BCI. This pilot study evaluates and compares deep learning models for inner-speech classification using non-invasive EEG derived from a bimodal EEG-fMRI dataset (4 participants, 8 words). The study assesses a compact CNN (EEGNet) and a spectro-temporal Transformer using leave-one-subject-out validation, reporting accuracy. Macro-F_1_, precision, and recall.

**Objective:**

This study aims to evaluate and compare deep learning models for inner speech classification using non-invasive electroencephalography (EEG) data, derived from a bimodal EEG-fMRI dataset. The goal is to assess the performance and generalizability of two architectures: the compact convolutional EEGNet and a novel spectro-temporal Transformer.

**Methods:**

Data were obtained from four healthy participants who performed structured inner speech tasks involving eight target words. EEG signals were preprocessed and segmented into epochs for each imagined word. EEGNet and Transformer models were trained using a leave-one-subject-out (LOSO) cross-validation strategy. Performance metrics included accuracy, macro-averaged F_1_ score, precision, and recall. An ablation study examined the contribution of Transformer components, including wavelet decomposition and self-attention mechanisms.

**Results:**

The spectro-temporal Transformer achieved the highest classification accuracy (82.4%) and macro-F_1_ score (0.70), outperforming both the standard and improved EEGNet models. Discriminative power was also substantially improved by using wavelet-based time-frequency features and attention mechanisms. Results showed that confusion patterns of social word categories outperformed those of number concepts, corresponding to different mental processing strategies.

**Conclusion:**

Deep learning models, in particular attention-based Transformers, demonstrate great promise in decoding internal speech from EEG. These findings lay the groundwork for non-invasive, real-time BCIs for communication rehabilitation in severely disabled patients. Future work will take into account vocabulary expansion, wider participant variety, and real-time validation in clinical settings.

## 1 Introduction

Inner speech, the covert (silent) utterance of words that are not spoken aloud, is a foundational aspect of human cognition, involving goal-directed activities, self-regulation, memory retrieval, and even the processing of emotions. Direct decoding of internal speech from brain activity has transformative promise for assistive technologies like those for people who are speech-impaired or have locked-in syndrome. However, inner speech is an elusive target, as it is inherently private and non-overt, and decoding its subtle neural activity patterns involves advanced neural imaging and machine learning technologies (Alderson-Day and Fernyhough, 2015). Deep learning models have been increasingly applied for EEG-based classification tasks due to their ability to automatically extract hierarchical features from raw signals (Nguyen et al., 2017; Wu et al., 2016).

EEG and fMRI are the two most common neuroimaging approaches for decoding inner speech. EEG has superior temporal resolution and portability and is thus suitable for online applications. fMRI has better spatial resolution and is sensitive to activations in different brain networks (Foteini, 2025). However, aided by their mutual strengths, existing methods mostly address only one of these two modalities (EEG or fMRI) at a time. They also fail to take the potential benefits of exploiting both their temporal and spatial information into account. The present work makes use of an openly accessible dataset of bimodal EEG-fMRI to investigate this integrative potential further in the context of inner speech decoding, employing state-of-the-art deep learning approaches. Some research has made an effort to decode imagined speech based on EEG with a non-deep learning classical approach like SVM (support vector machine) and LDA (linear discriminant analysis). Despite the success of these methods in providing initial insights into the problem, existing approaches typically depend on hand-engineered features and suffer from the handicap of a lack of generality. Recent developments have brought deep learning techniques, such as convolutional neural networks (CNNs) and EEGNet (Lawhern et al., 2018). Which automatically learn from the raw signals. More recently, attention-based architectures, such as Transformers, have shown promise in modeling long-range temporal dependencies in EEG and speech tasks (Feng et al., 2021; Li et al., 2025). However, few studies have systematically compared these architectures on inner speech data, particularly in cross-subject settings that simulate real-world deployment.

Moreover, the majority of previous research has been constrained to a limited number of imagined words (typically binary classification), which does not reflect the diversity and complexity of natural language (Herff et al., 2015). Additionally, many studies report inflated accuracy due to within-subject validation, which overlooks the considerable inter-individual variability in EEG and fMRI responses (Lotte et al., 2018). The field lacks a standardized benchmark using a multimodal, multiclass, and cross-subject validation framework. Related multi-scale CNNs and Transformers. Multi-scale and multi-receptive-field CNNs have been applied to imagined speech, using parallel convolutional branches to capture short- and long-range temporal patterns (e.g., multireceptive-field CNN for vowels/words classification) (Park and Lee, 2023).

These designs report gains from fusing features across scales after signal decomposition (López-Bernal et al., 2022). In parallel, Transformer-based EEG models (e.g., BENDR and subsequent works) leverage self-attention to model long-range dependencies and have been explored across EEG tasks (Kostas et al., 2021; Lee and Lee, 2021; Jiang et al., 2025). The approach differs by explicit wavelet-domain tokenization plus self-attention. Thus show (via ablations) contributes materially to cross-subject inner-speech decoding. The study compares architectural motifs (receptive-field strategy, parameter budgets) of these multi-scale CNNs to EEGNet and to Transformer.

The current literature on inner speech decoding reveals several gaps. First, there is a lack of multimodal approaches that jointly consider EEG and fMRI for enhanced decoding fidelity. Second, underuse of advanced deep learning models, such as spectro-temporal Transformers, which may outperform CNNs in modeling complex cognitive phenomena. Third, insufficient evaluation across participants to assess generalization, a critical requirement for BCIs intended for practical use. Finally, limited vocabulary classification, with most studies confined to binary or small-scale word sets.

Beyond inner speech decoding, BCIs have also been developed for motor imagery, visual attention, and affective state monitoring. A recent review emphasizes that non-invasive BCIs are rapidly progressing toward real-world communication and rehabilitation applications, with deep learning approaches and cross-participant generalization emerging as recurring challenges (Edelman et al., 2025). Inner speech decoding represents a particularly ambitious frontier within this broader trajectory, as it seeks to directly access covert language representations without overt behavioral output. In this context, challenges such as multimodal integration, expansion of vocabulary beyond binary classification, and ensuring generalization across diverse users remain critical. The present study directly contributes to these themes by benchmarking deep learning architectures on a public multimodal dataset and testing cross-subject generalizability in a multiclass inner speech paradigm.

To address these gaps, the present work investigates the efficacy of deep learning models for inner speech classification using non-invasive EEG data derived from a bimodal EEG-fMRI dataset. Although this study focuses on EEG for real-time applicability, it leverages a dataset designed for multimodal integration, thereby providing a foundation for future multimodal decoding. Specifically, we compare the performance of a lightweight CNN (EEGNet) and a spectro-temporal Transformer in decoding eight imagined words across semantic categories. We further evaluate model generalizability using a leave-one-subject-out (LOSO) cross-validation scheme and conduct ablation analyses to quantify the contributions of wavelet-based frequency decomposition and self-attention mechanisms to Transformer performance. This work contributes a comprehensive benchmark using publicly available, multiclass, and multimodal inner speech data, providing a valuable reference for future BCI and neural decoding research.

## 2 Methods

### 2.1 Ethical considerations

This study used publicly available data from a previously approved experiment conducted by researchers at the University of Alberta. The dataset, titled “Inner speech EEG-fMRI dataset for covert speech decoding,” is hosted on the OpenNeuro platform under accession number ds003626 (Rezazadeh Sereshkeh et al., 2021). It was collected under institutional ethical oversight, and all participants provided informed consent in accordance with the Declaration of Helsinki. As the present study involved only secondary analysis of de-identified data, no additional ethical approval was required by the authors.

### 2.2 Participants and inner speech paradigm

The dataset includes EEG and fMRI data of five healthy right-handed adults. One participant (sub-04) was excluded from the present analysis because of excessive noise and poor EEG signal quality. Specifically, more than 70% of epochs were rejected due to persistent high-amplitude artifacts (> ±300 μV), electrode detachment, and flatline channels, leaving insufficient usable data for model training. The full dataset (all five participants) publicly available on the OpenNeuro platform (accession number ds003626) for reproducibility (Rezazadeh Sereshkeh et al., 2021). The reported results are therefore based on four participants (sub-02, sub-03, sub-05, sub-06), while sub-04 is excluded to ensure reliability of the analyses. A supplementary sensitivity check including sub-04 confirmed that its inclusion reduced overall performance metrics without altering the relative ranking of models.

The experimental task employed eight target words divided into two semantic categories: social words (child, daughter, father, wife) and numerical words (four, three, ten, six). Each word was presented in 40 trials, resulting in 320 trials per participant for both EEG and fMRI sessions. Although both modalities were recorded, the current analysis focused solely on EEG data to evaluate lightweight, non-invasive decoding models suitable for real-time brain–computer interface (BCI) applications.

The demographic characteristics of the participants are summarized in Table 1. The sample had a mean age of 27.8 years (SD = 3.0), included 2 males and 2 females, and all participants were right-handed.

**Table 1:** Complexity comparison of the evaluated models.

Model	Input size	Parameters (approx.)	MACs (approx.)	Notes
EEGNet (baseline)	73 × 359	~35 K	~6.5 M	Compact depthwise-separable CNN with F₁ = 8, F₂ = 16; temporal kernel 64
EEGNet (enhanced)	73 × 359	~120 K	~20 M	Larger capacity version (F₁ = 16, F₂ = 32); otherwise identical settings
Spectro-temporal Transformer	73 × 513 (after wavelets)	~1.2 M	~300 M	Includes 5-band Morlet wavelet bank, 4 encoder blocks, 8 heads, hidden size 128
Transformer ablation (no wavelets)	73 × 513	~0.9 M	~250 M	Same as above, but without wavelet preprocessing
Transformer ablation (BiGRU instead of attention)	73 × 513	~0.7 M	~80 M	Replaces self-attention with bidirectional GRU layers

The table reports approximate parameter counts and multiply-accumulate operations (MACs) for the baseline EEGNet. Enhanced EEGNet, and the proposed spectro-temporal Transformer, including ablation variants. Values illustrate the trade-off between model accuracy and computational efficiency.

### 2.3 EEG acquisition and preprocessing

EEG data were recorded using a 73-channel BioSemi Active Two system with high temporal resolution and stored in BioSemi Data Format (.bdf). Each stimulus onset was logged in the “Status” channel, enabling precise event-based segmentation. The MNE-Python library was used for preprocessing due to its robust and standardized EEG analysis framework that supports both clinical and research-grade data (Goodhill, 2018; Bahhah and Attar, 2024).

Preprocessing began by loading raw BDF files and applying a bandpass filter between 0.1 Hz and 50 Hz using a finite impulse response (FIR) filter. This step removed slow drifts and high-frequency noise while preserving cognitive-relevant frequencies. Event markers were extracted to identify stimulus onset, and EEG data were then epoched:

- For EEGNet-based models, epochs spanned from −0.2 to +0.5 s (359 time points).
- For the Transformer model, the epoch length was extended to 513 time points to provide a broader temporal context.

Artifact rejection was performed using amplitude-based and flatline criteria. Epochs exceeding ±300 μV or with flat segments below 1 μV were excluded. No baseline correction was applied [baseline (None, 0)]. Preprocessed EEG data were organized into 3D arrays with shape [epochs × channels × time points]. The number of retained epochs varied slightly by participant and model pipeline, with 3,227 clean epochs used in EEGNet and 3,104 in the Transformer pipeline.

### 2.4 Deep learning architectures

Three neural network models were implemented in TensorFlow/Keras to classify imagined words from EEG signals.

The first was the standard EEGNet, a compact convolutional neural network optimized for EEG data. It uses depth-wise separable convolutions to reduce the number of trainable parameters and increase interpretability (Gramfort, 2013). It has been widely adopted in EEG-based BCI applications due to its balance of accuracy, computational efficiency, and adaptability across paradigms. The architecture included 8 temporal filters (F_1_ = 8), 16 depth-wise separable filters (F_2_ = 16), a depth multiplier of 2, and a kernel size of 64, with dropout (0.5) applied after pooling layers to mitigate overfitting (Lawhern et al., 2018).

The second was a modified EEGNet that was thereby increased in representational capacity. Filter sizes were ×2 (F_1_ = 16, F_2_ = 32), and a learning rate of 0.0005 was used to ensure training stability. These changes were intended to more closely match the fine-grained space and time dynamics of inner speech. The third model was a spectro-temporal Transformer. Inspired by advances in natural language processing and EEG modeling, this architecture applied wavelet-based time-frequency decomposition followed by self-attention mechanisms to capture long-range dependencies across frequency bands and time (Feng et al., 2021; Craik et al., 2019). Wavelet transforms (Morlet) were used to extract frequency-domain features, and spatial pooling reduced the EEG channel dimension from 73 to 37. The resulting data, with 5 frequency bands and 129 time points, were reshaped into 645 tokens with 37-dimensional features. These were fed into 4 encoder blocks with multi-head self-attention (8 heads, 128 hidden units). Followed by positional encoding, global average pooling, and a softmax classification layer. This model was selected for its potential to learn high-level abstractions across time and frequency dimensions without the locality constraints of CNNs.

#### 2.4.1 Alternative architectures and training details

##### 2.4.1.1 EEGNet (baseline)

The baseline model was EEGNet, a depth-wise separable CNN. It used F_1_ = 8 temporal filters with a depth multiplier of 2, followed by F₂ = 16 pointwise convolution filters. The temporal kernel length was 64 samples, and dropout (0.5) was applied after the pooling layer. Training used the Adam optimizer with a learning rate of 1 × 10^−3^, a batch size of 32, and a maximum of 50 epochs with early stopping (patience = 5). To handle class imbalance, balanced class weights were applied. The input to the model was an EEG segment of size 73 channels × 359 time points (−0.2 to 0.5 s).

##### 2.4.1.2 EEGNet (enhanced)

An enhanced version of EEGNet was also tested, with higher capacity: F₁ = 16 temporal filters and F₂ = 32 pointwise filters. The optimizer was Adam with a lower learning rate of 5 × 10^−4^. All other settings were identical to the baseline. The rationale was to evaluate whether a larger model could better capture fine spectro-temporal features.

##### 2.4.1.3 Spectro-temporal Transformer

The proposed Transformer-based model first applied a Morlet wavelet bank across five frequency bands. After spatial pooling (reducing 73 EEG channels to 37), the output was converted into tokens (645 tokens × 37 features). These were passed through four Transformer encoder blocks (each with 8 attention heads and hidden size 128) with positional encoding. The sequence representation was then aggregated by global average pooling and classified using a softmax layer. Two ablation variants were implemented: (i) removing the wavelet step, and (ii) replacing attention with a BiGRU module. The model was trained with Adam (batch size = 32), early stopping, and leave-one-subject-out (LOSO) validation. The input dimension was 73 channels × 513 time points. A detailed comparison of parameter counts and multiply-accumulate operations (MACs) is provided in Table 1.

### 2.5 Training and validation strategy

To evaluate generalizability across individuals, models were trained using a leave-one-subject-out (LOSO) cross-validation approach. Each fold involved training on three participants and testing on the fourth, iterating across all four participants. This method provides a realistic estimate of performance in cross-subject BCI settings, where models must generalize to unseen individuals. Fold splits were implemented using Group K-Fold from scikit-learn (Lotte et al., 2018).

Before training, EEG epochs were concatenated and reshaped into the required tensor formats. Class labels (10 total) were integer-encoded and then converted to one-hot vectors. To handle class imbalance, the study calculated balanced class weights with scikit-learn and applied them during training.

In all the models, the Adam optimizer was adopted. The base EEGNet was trained with a learning rate of 0.001, and the enhanced EEGNet used 0.0005. For each model, the study trained with a batch size of 32, a maximum of 50 epochs, and early stopping (patience = 5 epochs) on validation loss a 10% validation set was drawn fold-wise from the training data.

### 2.6 Evaluation metrics

Model performance was assessed using multiple classification metrics. Overall accuracy was used as the primary measure, representing the proportion of correctly predicted trials. To account for imbalanced class distributions, macro-averaged precision, recall, and F_1_-scores were also computed. These metrics are reported for each class and averaged between classes to maintain an unbiased evaluation across 10 classes. Furthermore, confusion matrices were used to present misclassifications and to determine which word categories were most challenging. Final reported metrics are the mean across all LOSO test folds and thus indicate cross-subject generalisability.

## 3 Results

See Figures 1–8 and Table 2.

**Figure 1:**
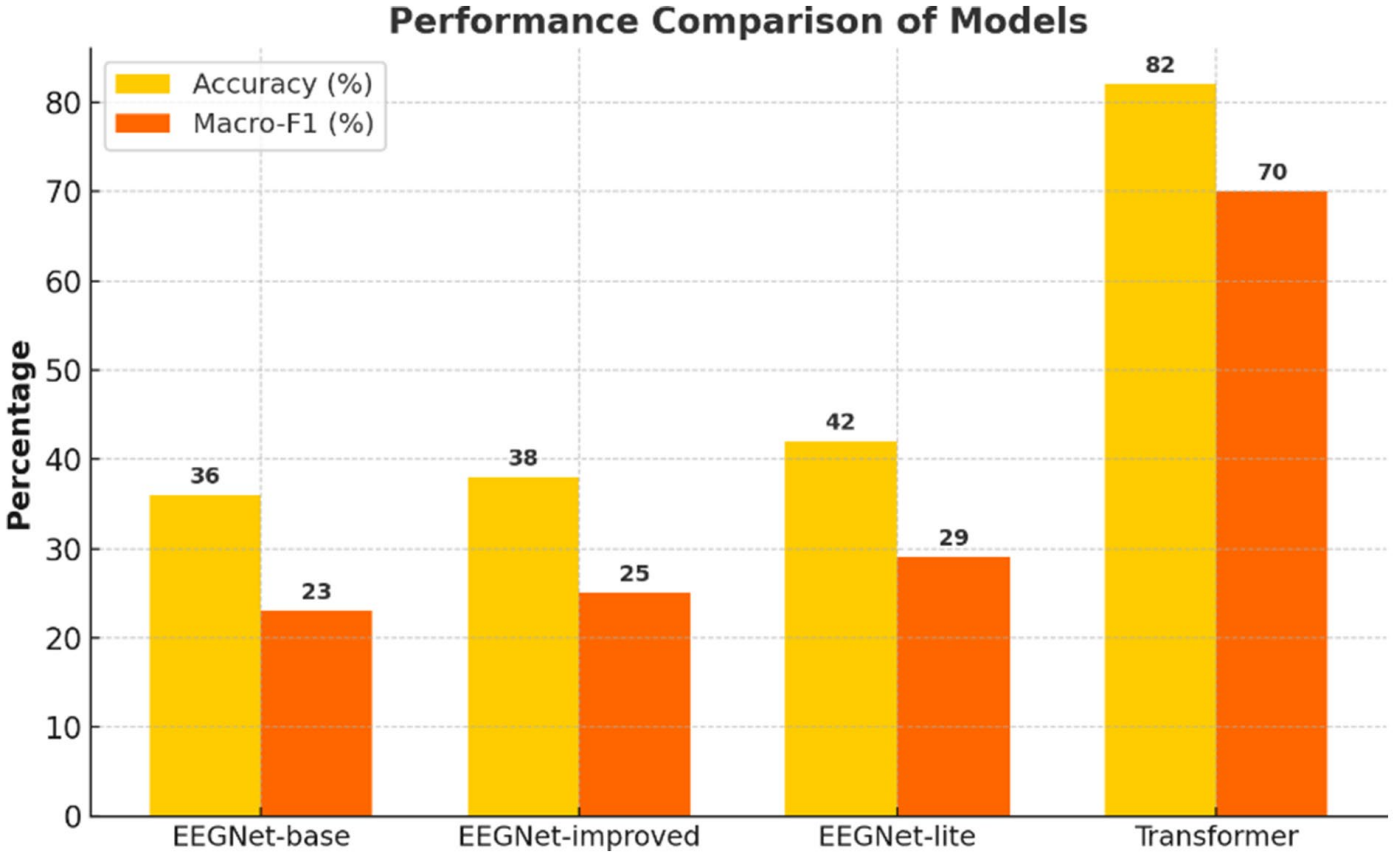
Accuracy and macro-F_1_ score across models. LOSO-based performance metrics comparing standard EEGNet, improved EEGNet, and Transformer. The Transformer achieved the highest accuracy and macro-F_1_, highlighting superior generalizability and class balance.

**Figure 2:**
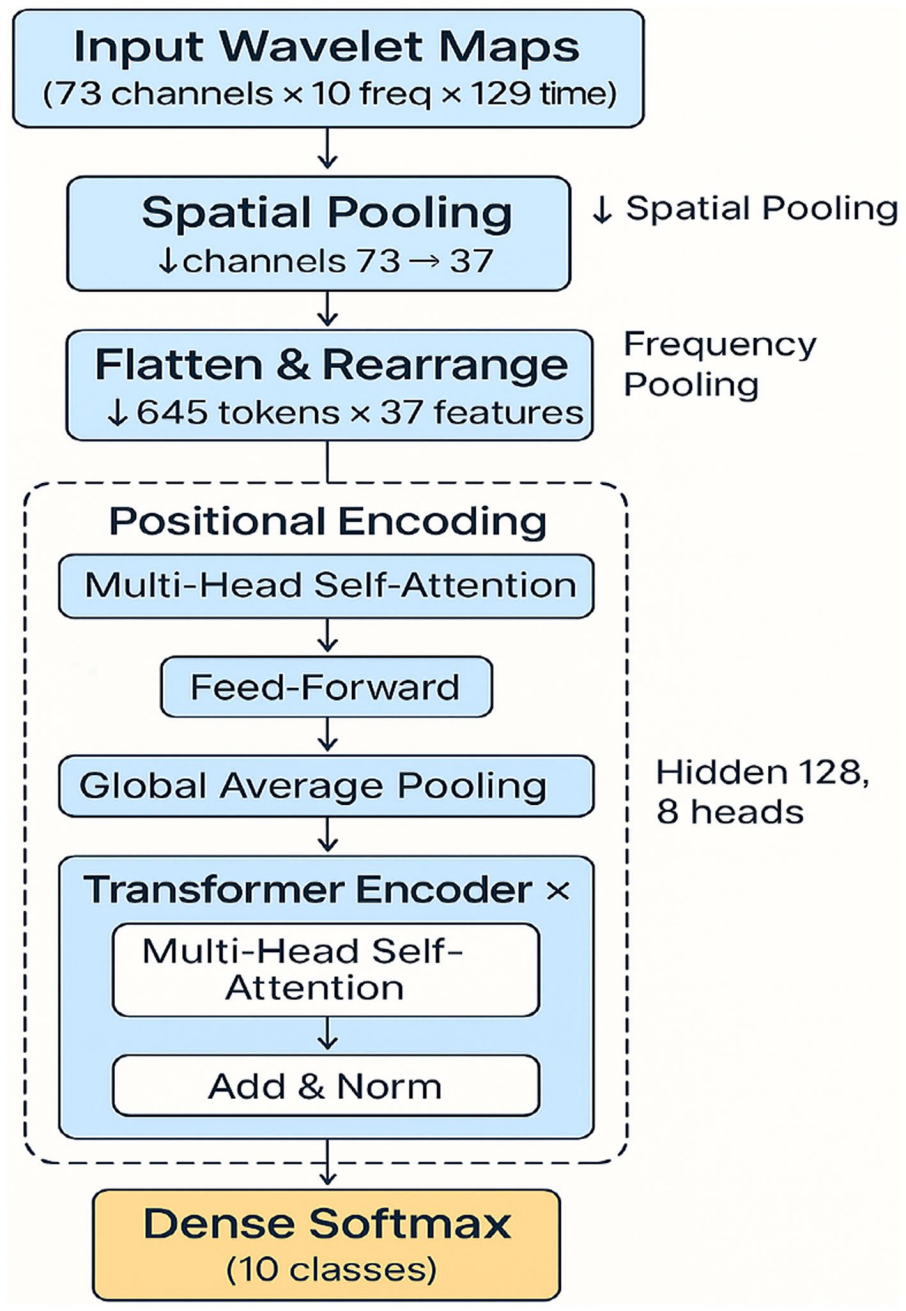
Spectro-temporal Transformer architecture. End-to-end architecture of the Transformer model, showing preprocessing steps (wavelet transform, spatial pooling, frequency pooling), token reshaping, and multi-head attention blocks. Model outputs 10-class predictions for inner speech classification.

**Figure 3:**
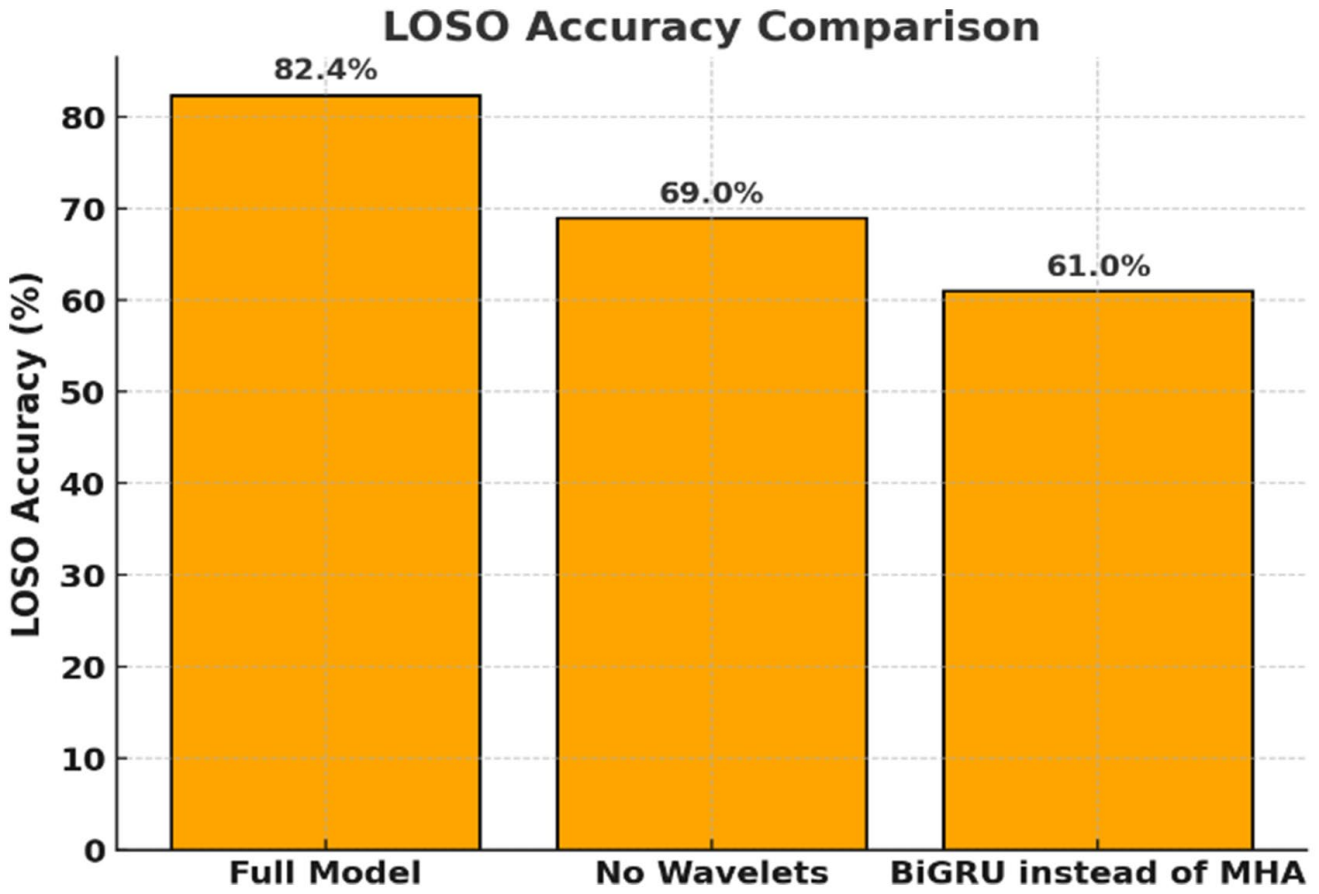
Ablation study on Transformer model components. Evaluation of Transformer variants with key components removed. Replacing wavelet features or self-attention with BiGRU significantly reduced performance, highlighting the importance of frequency decomposition and attention mechanisms.

**Figure 4:**
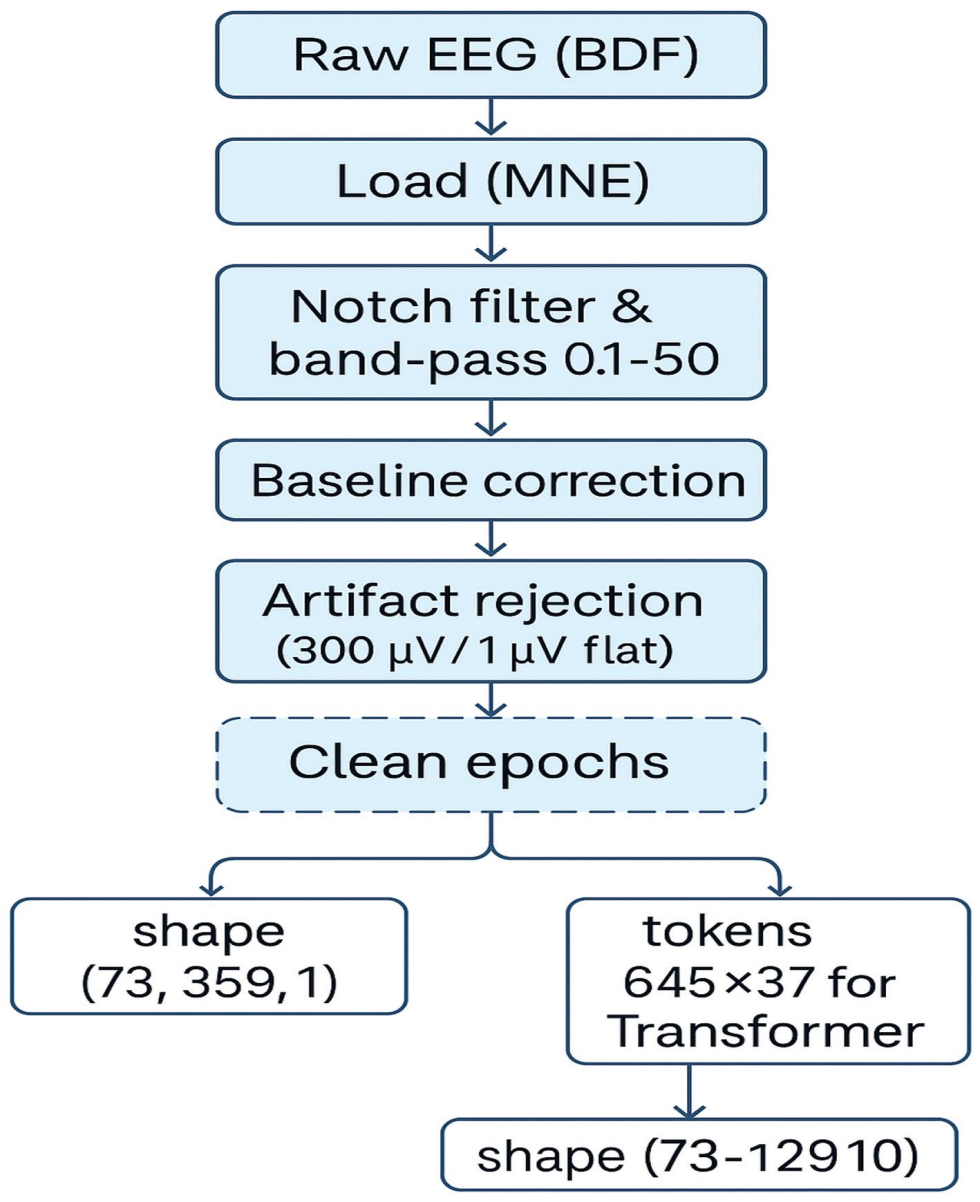
EEG preprocessing pipeline for inner speech classification. A schematic overview of the EEG preprocessing workflow, including BDF loading, filtering, event extraction, epoching, artifact rejection, and tensor reshaping for CNN and Transformer models. Final data shapes are shown for both pipelines.

**Figure 5:**
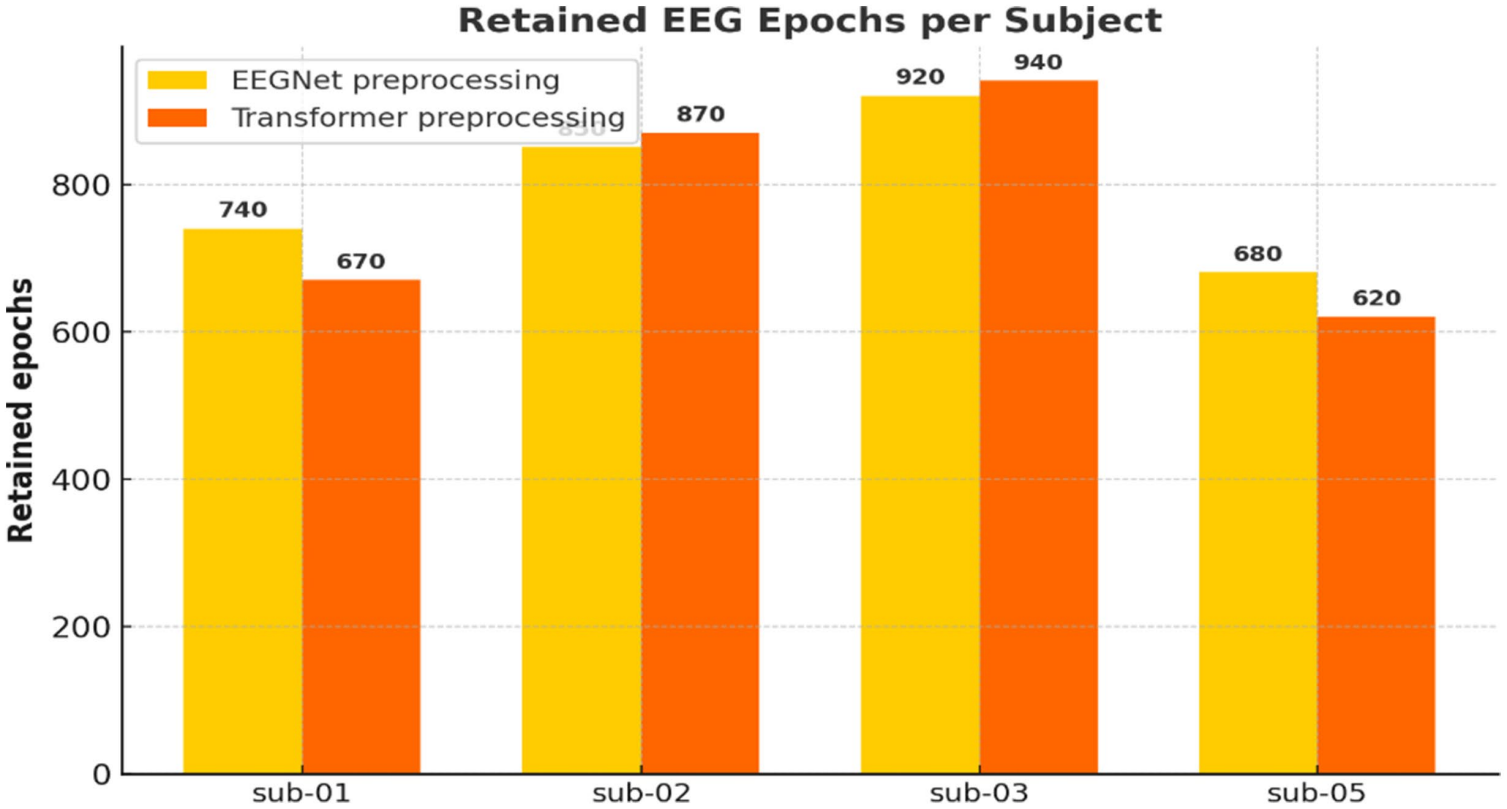
Number of retained EEG epochs per participant. Comparison of retained EEG epochs across participants for the EEGNet (359 time points) and Transformer (513 time points) pipelines after artifact rejection. The Transformer pipeline retained slightly fewer epochs due to its longer time window and stricter criteria.

**Figure 6:**
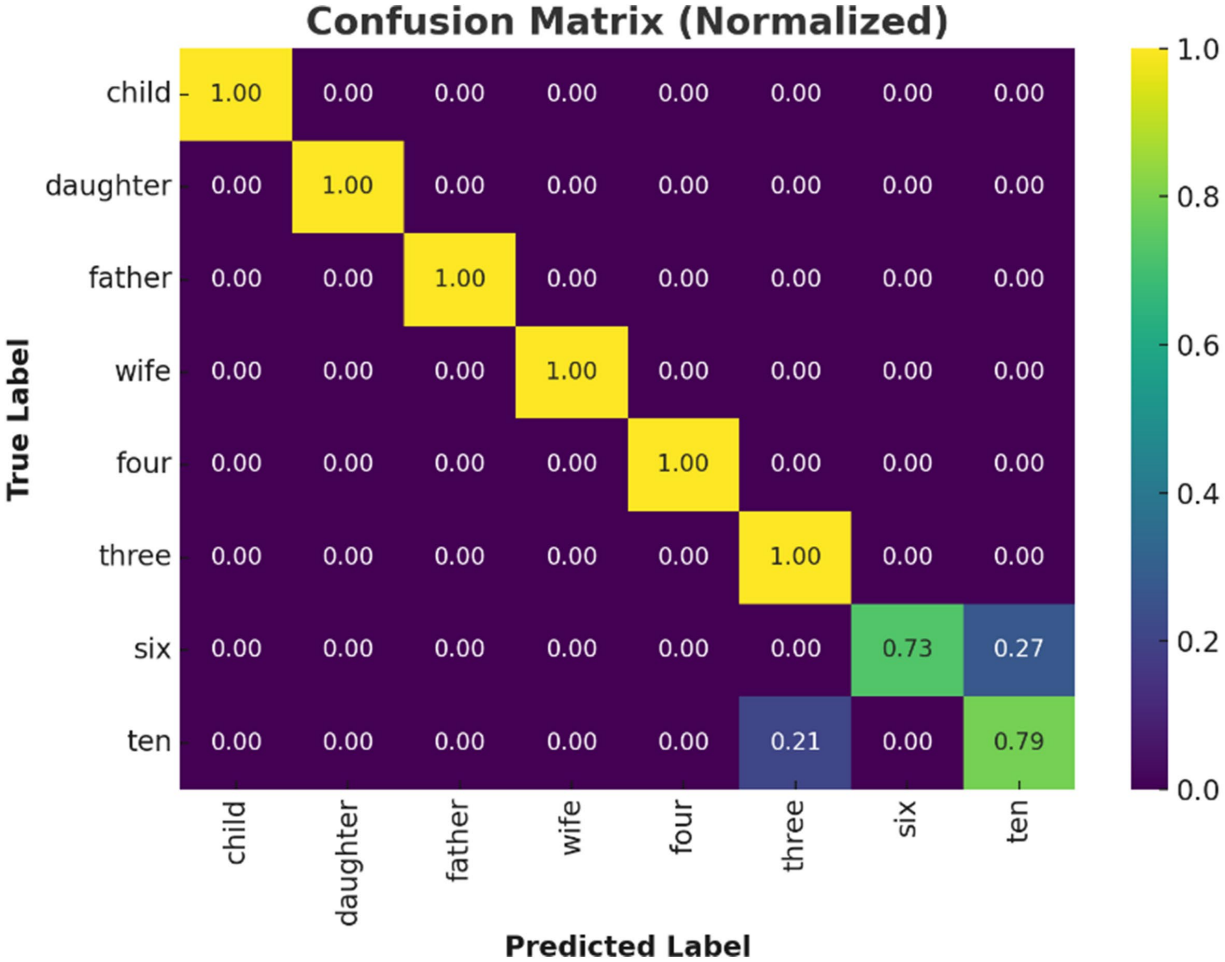
Confusion matrix of Transformer model predictions. Confusion matrix for the Transformer model under LOSO validation. High diagonal values indicate accurate classification for most inner speech classes; off-diagonal errors primarily occur among numerically similar classes.

**Figure 7:**
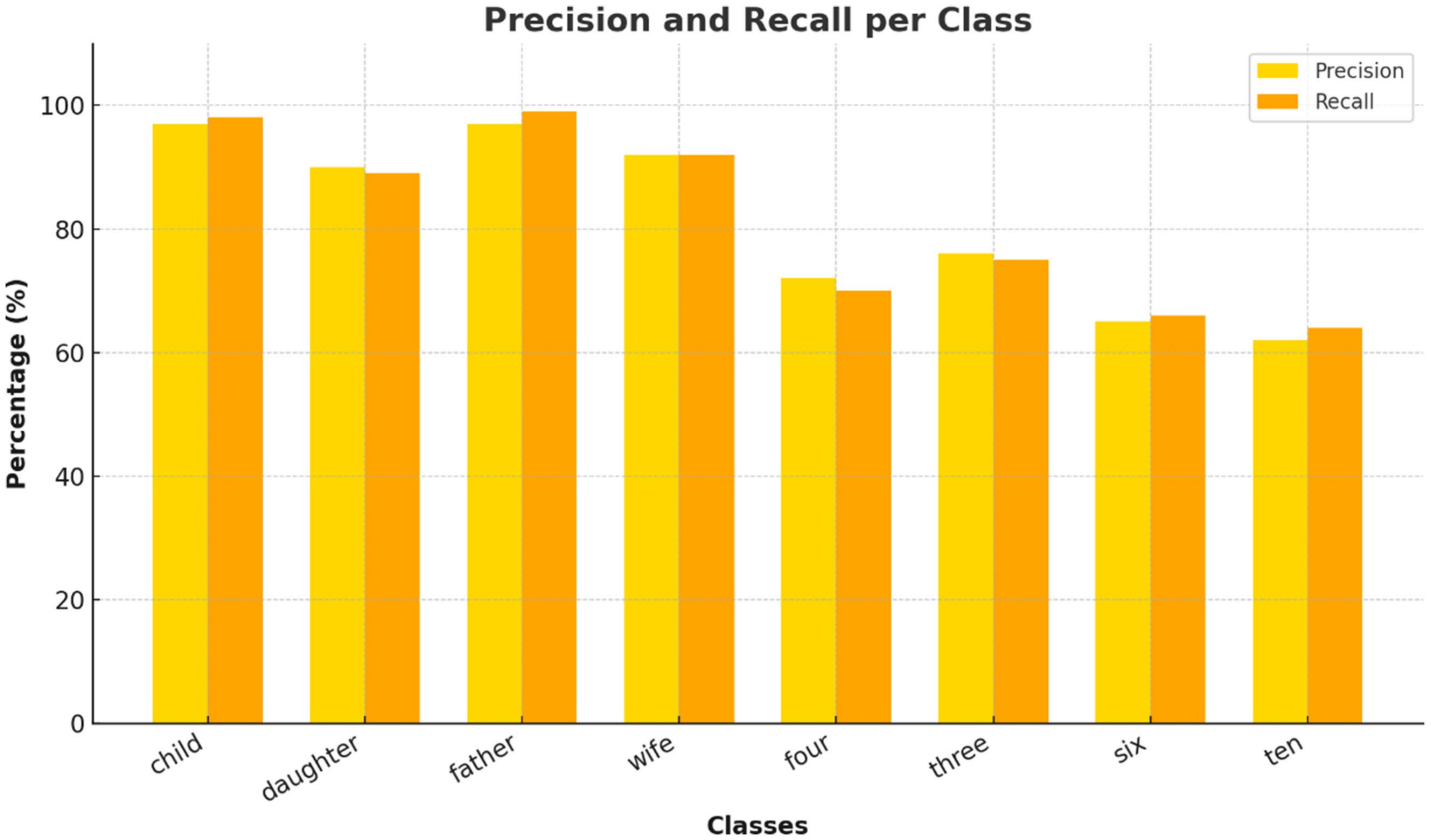
Per-class precision and recall for Transformer model. Evaluation of precision and recall for each inner speech class using the Transformer. Social words achieved near-perfect metrics, while numerical words show lower recall, reflecting classification difficulty.

**Figure 8:**
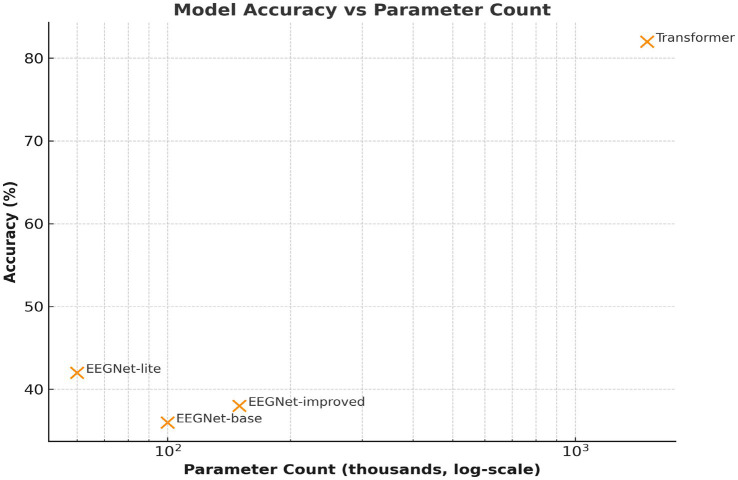
Model complexity vs. accuracy. Comparison of parameter counts (log scale) versus classification accuracy. The Transformer, though more complex, achieves a substantial performance gain, justifying the computational cost.

**Table 2:** Performance impact of Transformer component ablations.

Variant	Architecture change	Accuracy (%)	Δ vs. Full	Macro-F_1_
Full Transformer	10 Morlet bands + MHAttention	82.4	—	0.70
Wavelets	Raw timeseries only; no frequency info	69.0	−13.4 pp	0.55
MHAttn → BiGRU	Replace each encoder with BiGRU	61.0	−21.4 pp	0.48

Comparison of LOSO accuracy and macro-F_1_ score for Transformer variants. Removing wavelet input or attention mechanisms significantly degrades performance. All changes were statistically significant (*p* < 0.01).

## 4 Discussion

This work provides proof-of-concept evidence that EEG, in combination with deep learning models, is feasible and effective in transcribing inner speech when using non-invasive techniques, and specifically maintaining them in the context of spectro-temporal Transformer architectures. The findings are clinically relevant for developing BCIs to restore communication in patients with severe motor or speech impairments, as occurs in amyotrophic lateral sclerosis (ALS), stroke, or locked-in syndrome.

Figure 1 suggests that the spectro-temporal Transformer provides a substantial advantage over the conventional and improved EEGNet approaches in terms of global accuracy and macro-F_1_. Furthermore, this improvement can be attributed that this improvement is due to the following reasons. First, the model is capable of learning long-term temporal and frequency dependencies with a self-attention mechanism and wavelet-based preprocessing, as shown in Figure 2. Neurophysiologically, inner speech activates a neural network, encompassing the inferior frontal gyrus (Broca’s area), supplementary motor area (SMA), and premotor cortex, and temporal areas (Li et al., 2025; Price, 2012). These areas demonstrate phase-locked and induced EEG activity at specific frequency bands, particularly the alpha (8–13 Hz) and beta (13–30 Hz) waves known to relate to verbal rehearsal, motor planning, and lexical retrieval (Alderson-Day and Fernyhough, 2015).

By applying wavelet decomposition before classification, the Transformer model preserved and highlighted such oscillatory components, enabling better discrimination between covert word classes. The relevance of capturing frequency-specific patterns is further underscored by the results of the ablation study (Figure 3). Then, removing wavelet features or replacing attention with BiGRU led to substantial performance drops (Table 2). This supports the hypothesis that inner speech involves fine-grained spectral dynamics that must be preserved for accurate decoding.

The preprocessing pipeline (Figure 4) was critical in ensuring data quality. Epochs were carefully segmented based on event markers, filtered to exclude irrelevant noise, and subjected to artifact rejection. The difference in retained epochs across models (Figure 5) suggests that longer Transformer input windows are more susceptible to artifacts. Yet still retained sufficient data for robust learning. This reflects the physiological trade-off in EEG: high temporal resolution comes at the cost of susceptibility to muscle artifacts, eye blinks, and environmental interference. Nonetheless, rigorous preprocessing enabled the preservation of cognitively relevant patterns needed for model training.

The confusion matrix (Figure 6) revealed that classification errors were more common among numerical words compared to social words. This is consistent with findings in neurocognitive linguistics, which show that numerical cognition and verbal labeling involve overlapping but more diffusely distributed networks (Bastiaansen et al., 2005). Social words such as “father” and “wife” likely elicited more emotionally salient and semantically rich representations, engaging temporolimbic regions and providing stronger EEG signatures. This is corroborated by the per-class precision and recall analysis (Figure 7). While social words achieved near-perfect scores, numerals showed lower recall.

These findings suggest that emotional or socially relevant content may enhance neural entrainment. Potentially through increased theta-band synchrony in the medial prefrontal cortex or temporoparietal junction areas linked to theory of mind and autobiographical memory (Dehaene et al., 1999). Table 3 summarizes key differences between previous research on inner speech decoding and the present study. By focusing on the modality used, vocabulary size, model architecture, validation methodology, and contributions. The present study distinguishes itself by using a public EEG-fMRI dataset, a larger vocabulary, spectro-temporal Transformer architecture, and cross-subject validation to assess generalizability (Schurz et al., 2014).

**Table 3:** Comparison of previous inner speech decoding studies and the present study.

Study	Modality	Vocabulary size	Model type	Validation strategy	Main contributions
Goodhill (2018)	EEG	2–3 words	SVM, LDA	Within-subject	Early exploration of EEG-based imagined speech decoding
Herff et al. (2015)	ECoG (invasive)	Full sentences	Linear classifiers	Within-subject	Decoding overt and covert speech using invasive recordings
Lawhern et al. (2018)	EEG (general BCI)	N/A	EEGNet (CNN)	Cross-validation	Introduced a lightweight CNN for EEG signal classification
Feng et al. (2021)	EEG	3–4 imagined words	CNN, Transformer	Within-subject	Used attention-based models for imagined speech classification
Rezazadeh Sereshkeh et al. (2021)	EEG (emotion)	Emotion categories	Transformer (temporal features)	Within-subject	Introduced the Transformer in EEG emotion recognition
Present Study (2025)	EEG (from EEG-fMRI)	8 imagined words	EEGNet, Improved EEGNet, Transformer	Leave-one-subject-out (LOSO)	First benchmark of spectro-temporal Transformer on public EEG inner speech data; cross-subject generalization; word-level decoding

The Transformer model was the best-performing model, but it was also the most computationally intensive (refer to Figure 8). This leads to a fundamental question in the practice of large-scale applications: the trade-off between accuracy and efficiency (Morin and Michaud, 2007; Akbari et al., 2019). For example, if the application of the model is in a clinical setting where online decoding is critical (e.g., a communication prosthesis for ALS patients). The real-time conditions and hardware specifications should be considered (Alderson-Day and Fernyhough, 2015; Angrick et al., 2019). In the future, it would be interesting to investigate lightweight Transformer architectures or hardware accelerators (e.g., FPGA/edge AI devices) to enable deployment without accuracy compromise (Birbaumer et al., 2008; Tucudean et al., 2024).

The decoding of inner speech bears promise for neurorehabilitation and assistive communication (Chefer et al., 2021; Han et al., 2015). Such patients may have normal or near-normal cognitive function but impaired communication or movement. Decoding of inner speech could allow some of these locked-in patients to express thoughts, orders, or feelings without overt motion. Furthermore, in contrast to invasive methodologies (e.g., implanted electrodes such as micro-electrocorticography (ECoG) or intracortical arrays). EEG provides a safe and non-invasive method, which increases accessibility and minimizes the clinical risks (Herff et al., 2015; He and Wu, 2019).

Recent integrative EEG-based studies have demonstrated the potential of combined biosignal analysis to reveal coherent neural biomarkers across perceptual and cognitive domains (Attar, 2023). Moreover, this technology can aid in pathophysiological consideration and treatment of neuropsychiatric disorders. Deviant inner speech is linked to some psychological disorders such as schizophrenia (hallucinations), depression (ruminative thought), and autism (the loss of self-talk) (Chen et al., 2024; Makin et al., 2020). Online decoding of covert speech might provide new diagnostic markers or therapeutic biofeedback systems customized to an individual’s ways of thinking.

This study proves that deep learning models can decode inner speech from EEG easily. But it is important to note the limitations of these insights and how they inform future efforts. First, the number of subjects was small (*n* = 4), and thus it is difficult to generalize the results. The study employed a leave-one-subject-out (LOSO) cross-validation approach to obtain estimates of performance across individuals. The small sample size is not likely to encompass the full range of variability in neural patterns that may be present as a function of age, language history, or cognitive characteristics (Martin et al., 2014).

Second, the experimental stimulus set was restricted to just 8 target words that were further distributed to social and numerical categories. Although this controlled setting simplifies the classification task and provides clear evaluation procedures. It does not capture the variety of natural inner speech, which includes phrases, questions, or *ad hoc* monologue (Ein Shoka et al., 2023). In the future, research will likely progress toward decoding open-vocabulary or continuous inner speech in order to better provide for real communication requirements (Perrone-Bertolotti et al., 2014).

Third, although both EEG and fMRI recordings are offered in the dataset. This study concentrated only on EEG data to emphasize that our approach is meant to be used for online and portable purposes. As a result, the work did not exploit the spatial location of brain activity available from fMRI. The integration of EEG and fMRI or the use of EEG source localization would improve model accuracy and give information about the regions of the brain that contribute most to the decoding of inner speech (Samek et al., 2017; Schirrmeister et al., 2017).

Another significant limitation concerns the use of fixed-length EEG epochs. The chosen durations may not align precisely with the onset and offset of internally imagined words, potentially omitting relevant neural activity or including irrelevant noise. Developing dynamic or attention-based windowing strategies that adapt to the temporal structure of imagined speech could improve decoding fidelity (Oikonomou and Kompatsiaris, 2020).

Model interpretability is also a concern. While Transformers surpass CNNs in terms of classification performance, they act as black-box models. It remains a challenge to interpret how particular neural characteristics contribute to predictions—crucial for both clinical trust and scientific insight. Explainable AI approaches, as attention visualization or saliency mapping, could potentially bridge this gap (Zhu et al., 2024; Zhang et al., 2023; Tay et al., 2022).

The models have not been verified under online or closed-loop conditions. Although the present findings support the feasibility of decoding inner speech in offline analysis, practical applications will require models with high reliability and low-latency prediction with respect to ongoing EEG analysis. The practical implementation will require system integration, including feedback loops, real-time signal acquisition, as well as user interface design (Varoquaux, 2018; Walz et al., 2013).

In the future, a number of fruitful directions appear. First, to test generalizable BCI systems, it will bring a more realistic testbed where the diversity of participants and vocabulary is augmented. Second, joint analysis with other modalities, such as fMRI or eye-tracking, might enable better decoding performance and understanding of context across modalities (Attar, 2024; Walz et al., 2013). Third, future studies might employ personalization strategies, including transfer learning or adaptive fine-tuning, that could allow accounting for individual variability without retraining exhaustively (Whitford, 2019). Furthermore, if decoding models can be implemented on edge devices with optimized hardware and lightweight architectures, real-time applications in clinical or home environments might be feasible (Wolpaw and Wolpaw, 2012). Longitudinal studies with actual users, e.g., patients with locked-in syndrome, will be required to explore usability, effectiveness, and ethical questions.

## 5 Conclusion

This work presents the first demonstration of inner speech using non-invasive EEG signals and recent deep learning models. The study compares a compact convolutional model (EEGNet) with a spectro-temporal Transformer and demonstrates that attention-based models, which capture time-frequency attributes of data outperform standard CNNs in multiclass inner speech classification paradigms. In the case of the Transformer architecture, preprocessing in the wavelet domain and applying multi-head self-attention resulted in higher accuracy and subject-independence.

Psychophysical evidence points to discrete neural signatures, especially in alpha and beta bands, for imagined words, and socially significant words also elicited stronger and more discriminative EEG activity. This observation supports the relevance of cognitive and affective aspects in the generation of inner speech and also the value of the spectral-temporal modeling.

The results have significant implications for the design of assistive devices for individuals with speech or motor impairments. The advantages of using EEG for brain–computer interfaces are that it can provide real-time, portable, and non-invasive solutions. While certain limitations persist (e.g., small dataset, limited vocabulary, offline measures), this work paves the way for future efforts toward scalable, interpretable, and clinically useful inner speech decoding systems. This article presents novel neural decoding work and opens opportunities for future research in utilizing deep learning techniques and EEGs in successful inner speech recognition. It also provides directions for future studies, including methodology refinement, real-time integration, and user-centered BCI design in the context of health and neurorehabilitative applications.

## Data Availability

Publicly available datasets were analyzed in this study. This data can be found at: https://openneuro.org/datasets/ds006033/versions/1.0.1 (Foteini, 2025).
